# Total knee arthroplasty in patients with Parkinson’s disease: A systematic review and meta-analysis protocol

**DOI:** 10.1097/MD.0000000000032315

**Published:** 2022-12-30

**Authors:** Guangchen Sun, Hui Yu, Jun Cui, Ming Li, Yuefang Ru

**Affiliations:** a Department of Orthopedics, the First People’s Hospital of Jiashan, Zhejiang Province, China.

**Keywords:** function, meta-analysis, outcome, Parkinson’s disease, total knee arthroplasty

## Abstract

Parkinson’s disease (PD) patients have been shown to have various musculoskeletal problems, the postoperative outcomes of total knee arthroplasty procedure might be less predictable if performed on a patient who has PD. We conducted a protocol for systematic review and meta-analysis to evaluate the functional outcomes, activity levels, mortalities, implant survival rates, and complications of total knee arthroplasty in patients with PD.

**Methods:**

This study follows the guideline of the preferred reporting items for systematic reviews and meta-analyses protocols and has been registered on the International Prospective Register of Systematic Reviews with CRD42022375885. Two independent reviewers will search for databases including PubMed, Embase, Cochrane Library website, ClinicalTrials.gov databases, Chinese National Knowledge Infrastructure Database, Wanfang database, and VIP database using the search strategies recommended by the Cochrane Back Review Group. The RevMan 5.3 software (Cochrane Collaboration, Oxford, UK) will be used to conduct the meta-analyses.

**Results:**

The results of this systematic review will be published in a peer-reviewed journal.

**Conclusion:**

This study may provide evidence for the clinical application of total knee arthroplasty in patients with PD.

## 1. Introduction

Total knee arthroplasty (TKA) is an efficacious surgical treatment for advanced knee osteoarthritis which could reduce pain and maintain motor function.^[1–3]^ The number of TKAs performed has increased substantially, and future demand is projected to rise rapidly. The rise in incidence of osteoarthritis has resulted in an increase in the total annual volume of TKA in the United States. By 2030, the demand for primary TKA is projected to grow to 3.48 million procedures.^[4]^ The quest to improve patient outcomes is never-ending. Therefore, the imminent demand for TKA reinforces the importance of analyzing indications, patient populations, comorbidities, and outcomes.^[5]^

Parkinson’s disease (PD) is a neurodegenerative disorder affecting millions of people worldwide.^[6,7]^ The disease is caused by the decrease in the dopaminergic activity in the nigro striatal pathway, leading to not only classical motor symptoms such as rigidity, bradykinesia, tremor, and postural instability, but also a range of non-motor features including autonomic, cognitive, psychiatric, and behavioral dysfunctions.^[8,9]^ It is reasonable to expect that as PD progresses, most patients undergoing TKA will experience severe physical disabilities and complications which may impair their ability to live independently.^[10,11]^ Therefore, it is of paramount importance for the orthopedic surgeon to have clarity and knowledge in the approach of TKA in PD. Although a few studies have reported on TKA results in PD patients, long-term follow-up studies of TKA in PD patients are rarely reported, and its clinical and radiologic outcomes remain controversial.

In this study, we conducted a protocol for systematic review and meta-analysis to compare the functional outcomes, activity levels, mortalities, implant survival rates, and complications of TKA in patients with PD with those in patients in a control group. Once these predictors are known, we should attempt to avoid them whenever possible to minimize the risk of complications after primary TKA implantation.

## 2. Methods

### 2.1. Ethics and registration

This is a literature-based study, thus ethical approval are not required. The protocol of this study follows the guideline of the preferred reporting items for systematic reviews and meta-analyses protocols^[12]^ and has been registered on the International Prospective Register of Systematic Reviews with CRD42022375885.

### 2.2. Inclusion criteria for study selection

The inclusion criteria are as follows: Type of studies: observational studies that have tested clinical efficacy and safety of TKA in PD patients. Publications in English and Chinese will be included; Type of participant: patients diagnosed with PD undergoing TKA will be included. There will be no restrictions with respect to gender, age, ethnicity or course of disease; Type of interventions: all patients in intervention group and control group receive TKA. There will be no restrictions with respect to implant type or surgical approach; Type of outcome measurements: the major outcomes include visual analogue scale, functional outcomes, and activity levels; the secondary outcomes include the incidence of deep venous thrombosis, pulmonary embolism and deep infection of knee joint.

### 2.3. Search strategy

Two independent reviewers will search for databases including PubMed, Embase, Cochrane Library website, ClinicalTrials.gov databases, Chinese National Knowledge Infrastructure Database, Wanfang database, and VIP database using the search strategies recommended by the Cochrane Back Review Group. The English search terms included following: “TKA” and “PD” with the Boolean logic operator “AND” and “OR.” Furthermore, reference cited in the relevant literature and other articles in the meta-analysis will be also reviewed. Table 1 provides the search strategies in the PubMed database, and other databases will use these strategies similarly.

**Table 1:** Search strategy for PubMed.

Number	Search terms
#1	Total knee arthroplasty [Ti/Ab]
#2	Total knee replacement [Ti/Ab]
#3	Joint arthroplasty[Ti/Ab]
#4	Joint replacement [Ti/Ab]
#5	Knee prosthesis [Ti/Ab]
#6	Replacement surgery [Ti/Ab]
#7	Arthroplasty surgery [Ti/Ab]
#8	Prosthesis implantation [Ti/Ab]
#9	#1 OR #2 OR #3 OR #4 OR #5 OR #6 OR #7 OR #8
#10	Parkinson’s disease [Ti/Ab]
#11	PD [Ti/Ab]
#12	Tremble [Ti/Ab]
#13	Shaking palsy [Ti/Ab]
#14	#10 OR #11 OR #12 OR #13
#15	#9 AND #14

Ab = abstract, Ti = title.

### 2.4. Data collection and analysis

#### 2.4.1. Selection of studies

**Figure 1.**
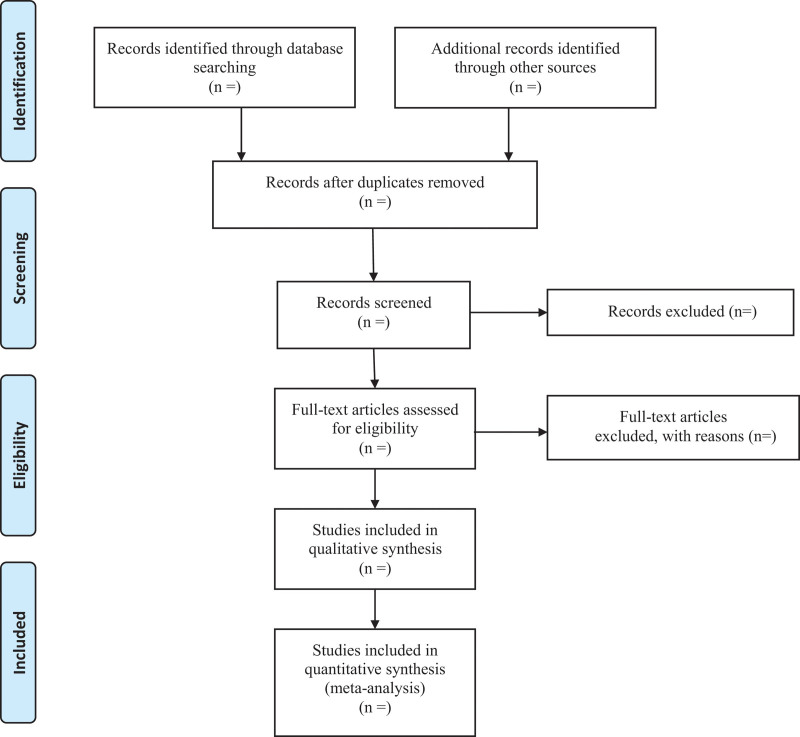
Flowchart of the study screening process.

Two researchers will independently remove the repeated data from the retrieved trails using the literature management system of EndnoteX7. They will then exclude the studies that are obviously not up to the standard by reading the titles and abstract, and decide the final inclusion of literature by reading the full text, group discussion, and contacting with the author to learn about the details of related researches. The final list of included references will be imported to a Microsoft Excel form. Both the literature search and the literature screening will be independently carried out by 2 research members. Another researcher will resolve the inconsistencies and check the last included literature should the need arise. The process of studies selection is presented in a PRISMA flow diagram (Fig. 1).

#### 2.4.2. Data extraction.

Two researchers will extract the data using the software EpiData 3.1. Another researcher will examine whether the extracted data are consistent and confirm the final retrieved data, including diagnosis, comorbidity, course, stage and severity of disease, sample size, age, sex, intervention, follow-up, outcome indicators, and adverse events. The research members will discuss and contact the authors for missing data, errors and/or uncertainties, or settle the related disputes by third party arbitration.

#### 2.4.3. Assessment of risk of bias

Two independent reviewers evaluated the methodological quality of the included clinical trials using Newcastle-Ottawa Scale (NOS). NOS scale is a tool for quality assessment of case-control studies and cohort studies. It is mainly evaluated based on three domains: selection of study cohorts, comparability of the cohorts, and outcome ascertainment. The total score is 9; it had high quality when NOS scores ≥ 6. Referring to the guidelines for assessing the risk of bias in the Cochrane Handbook for Systematic Reviews of Interventions, version 5.1.0,^[13]^ we conducted a bias analysis of the included studies. In case of disagreement and inconsistency, the researchers will discuss in the group, contact the author to determine the details, and seek third party arbitration.

#### 2.4.4. Assessment of heterogeneity

Chi-squared test (α = 0.1) and *I*^2^ value will be adopted respectively to analyze and determine the heterogeneity of the results of included researches. If *I*^2^ ≤ 50%, it can be deemed that the statistic heterogeneity among trials is negligible, and the fixed effects model will be employed to calculate the effect sizes. Otherwise the heterogeneity among the trials can be considered significant and random effects model will be used.

#### 2.4.5. Data synthesis

The RevMan 5.3 software (Cochrane Collaboration, Oxford, UK) will be used to conduct the meta-analyses. The difference of continuous variables in each study will be estimated using mean difference. The standardized mean difference will be used if continuous variables are large or are expressed using different units. The risk ratio and the corresponding 95% confidence interval will be used for the dichotomous variables. Sensitivity analyses will be evaluated by removing studies with high risk of bias or excluding one-by-one. Publication bias was evaluated by funnel plots and Egger test.^[14]^

## 3. Discussion

PD patients have been shown to have various musculoskeletal problems, which can impact their quality of life, such as osteoporosis, arthritis, fractures, and poor bone quality.^[15–17]^ These musculoskeletal problems can make orthopedic management of PD patients particularly challenging, especially as the patients get older and their bone quality further decreases.^[18]^ The postoperative outcomes of TKA procedure might be less predictable if performed on a patient who has PD due to the additional musculoskeletal problems associated with this disease.^[19]^ However, there is lack of sufficient evidence regarding the postoperative outcomes. This is the first meta-analysis to compared clinical efficacy and safety of patients with PD and controls after TKA. Higher quality clinical trials with large sample sizes are required for further research.

## Author contributions

**Conceptualization:** Hui Yu.

**Methodology:** Jun Cui.

**Software:** Ming Li.

**Writing – original draft:** Guangchen Sun.

**Writing – review & editing:** Yuefang Ru.
